# Molecular basis for allosteric specificity regulation in class Ia ribonucleotide reductase from *Escherichia coli*

**DOI:** 10.7554/eLife.07141

**Published:** 2016-01-12

**Authors:** Christina M Zimanyi, Percival Yang-Ting Chen, Gyunghoon Kang, Michael A Funk, Catherine L Drennan

**Affiliations:** 1 Department of Chemistry, Massachusetts Institute of Technology, Cambridge, United States; 2 Department of Biology, Howard Hughes Medical Institute, Massachusetts Institute of Technology, Cambridge, United States; 3 Center for Environmental Health Sciences, Massachusetts Institute of Technology, Cambridge, United States; Cold Spring Harbor Laboratory, United States

**Keywords:** X-ray crystallography, nucleic acid metabolism, allosteric regulation, *E. coli*

## Abstract

Ribonucleotide reductase (RNR) converts ribonucleotides to deoxyribonucleotides, a reaction that is essential for DNA biosynthesis and repair. This enzyme is responsible for reducing all four ribonucleotide substrates, with specificity regulated by the binding of an effector to a distal allosteric site. In all characterized RNRs, the binding of effector dATP alters the active site to select for pyrimidines over purines, whereas effectors dGTP and TTP select for substrates ADP and GDP, respectively. Here, we have determined structures of *Escherichia coli* class Ia RNR with all four substrate/specificity effector-pairs bound (CDP/dATP, UDP/dATP, ADP/dGTP, GDP/TTP) that reveal the conformational rearrangements responsible for this remarkable allostery. These structures delineate how RNR ‘reads’ the base of each effector and communicates substrate preference to the active site by forming differential hydrogen bonds, thereby maintaining the proper balance of deoxynucleotides in the cell.

**DOI:** http://dx.doi.org/10.7554/eLife.07141.001

## eLife digest

DNA contains the instructions required to make proteins and other molecules in cells. DNA is made of four building blocks called deoxyribonucleotides, which are in turn made from molecules called ribonucleotides by enzymes known as ribonucleotide reductases (RNRs for short). RNR enzymes are responsible for maintaining a good balance in the levels of the different deoxyribonucleotides in cells, which is essential for DNA to be made and repaired correctly.

Previous work has shown that each RNR can act on all four ribonucleotides. However, these enzymes become more selective for certain ribonucleotides depending on which deoxyribonucleotide is most common within the cell. For example, when a deoxyribonucleotide called dGTP is plentiful, it binds to a so-called “specificity site” on the enzyme and alters the shape of the enzyme’s active site. This then means that a ribonucleotide called ADP will bind in preference to the other ribonucleotides. However, it was not clear how the binding of deoxyribonucleotides to the enzyme influences the shape of the active site.

Zimanyi et al. used a technique called X-ray crystallography to determine the three-dimensional structures of a bacterial RNR enzyme when it is bound to all four different combinations of deoxyribonucleotides and ribonucleotides. In the absence of nucleotides, the active site adopts a shape that resembles an open barrel. However, when RNR is bound to a deoxyribonucleotide at the specificity site and a ribonucleotide at the active site, the barrel clamps down, bringing the specificity site and the active site closer together. Additionally, a loop of the protein interacts with each of the deoxyribonucleotides in a different way and communicates their identity directly to the active site, which rearranges itself to hold on to the corresponding preferred ribonucleotide.

Zimanyi et al.’s findings provide an explanation for how RNRs can select between ribonucleotides so that they produce a good balance of deoxyribonucleotides in cells. This will inform future efforts to develop molecules that inhibit RNRs, which may have the potential to be used to treat bacterial infections or to kill cancer cells.

**DOI:** http://dx.doi.org/10.7554/eLife.07141.002

## Introduction

**Figure 1.**
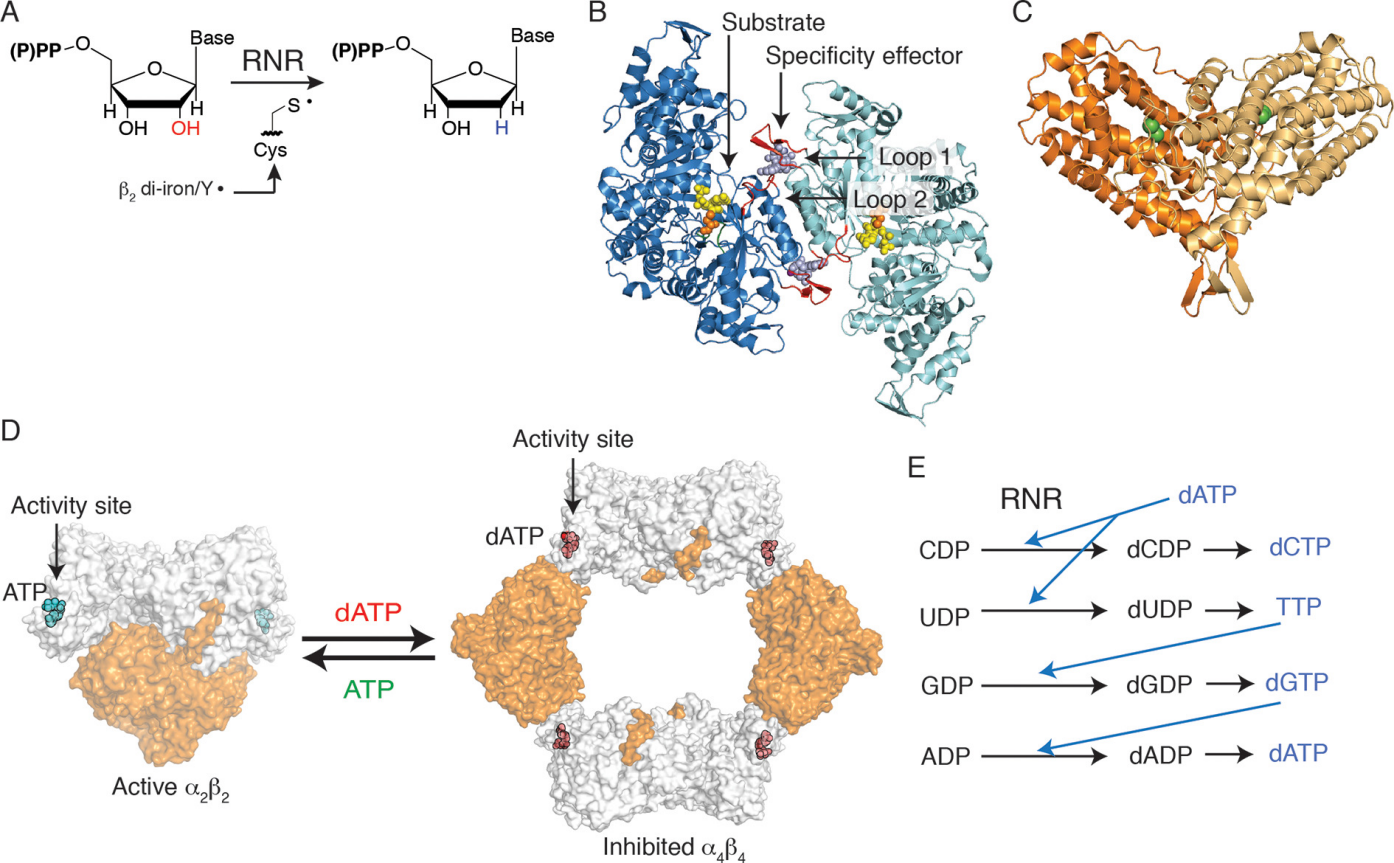
*Escherichia coli* class Ia RNR regulation is achieved through allostery. (**A**) *E. coli* RNR catalyzes reduction of nucleoside diphosphates using a radical, formed on an active site cysteine, to initiate catalysis. (**B**) A ribbon representation of the catalytic subunit (α_2,_ a 172-kDa homodimer) is shown with one α chain colored blue and the other cyan (this work). Nucleotides are shown as spheres with NDP substrate in yellow and dNTP specificity effector in purple. Loop 1 and loop 2, which are involved in specificity effector binding and recognition, are colored in red. Cys439, where the active site thiyl radical is formed, is shown in orange spheres. (**C**) A ribbon representation of the radical generating subunit (β_2_, an 87-kDa homodimer) is shown with one β chain colored orange and the other tan (this work). The di-iron cofactor that generates the initial tyrosyl radical required for RNR activity is shown in green spheres. (**D**) Allosteric activity regulation is achieved by interconversion between an active α_2_β_2_ complex in the presence of the allosteric activity effector ATP and an inactive α_4_β_4_ species when dATP binds to the allosteric activity site (PDB ID: 3UUS). The model for the α_2_β_2_ complex was created using small-angle X-ray scattering data (Ando et al., 2011) to fit the previously solved structure of α_2_ (PDB ID: 3R1R) and β_2_ (PDB ID: 1RIB) together. The α_2_ subunit is shown in grey surface representation, at a 90° angle from the representation shown in (B) and the β_2_ subunit is shown in orange surface representation. Allosteric activity sites are shown with ATP modeled in cyan and dATP in red spheres. (**E**) Allosteric specificity regulation is governed by the binding of deoxynucleoside triphosphates to RNR, influencing the preference for one substrate over another (see Table 1). **DOI:** http://dx.doi.org/10.7554/eLife.07141.003

Deoxyribonucleotides, the building blocks for DNA biosynthesis, are produced in the cell from ribonucleotide precursors by ribonucleotide reductase (RNR) (Figure 1). Three classes of RNRs are known, categorized by the cofactor they use to generate a protein radical required for catalysis. The best characterized of all RNRs is the class Ia enzyme from *Escherichia coli* that employs a di-iron-tyrosyl-radical cofactor to initiate chemistry and requires two dimeric protein subunits for enzymatic activity. The α_2_ subunit contains two (β/α)_10_ barrels, which house the active sites at the barrel centers (Eriksson et al., 1997; Uhlin and Eklund, 1994), and the β_2_ subunit utilizes a largely helical secondary structure to house the radical cofactor (Sjöberg and Reichard, 1977) (Figure 1B–C). As a central controller of nucleotide metabolism, RNR uses multiple allosteric mechanisms to maintain the balanced deoxyribonucleoside triphosphate (dNTP) pools that are required for accurate DNA replication. First, allosteric activity regulation modulates the overall size of dNTP pools. ATP or dATP binding at an allosteric activity site, found at the N-terminus of α_2_ (Figure 1D), leads to up-regulation or down-regulation of enzyme activity, respectively (Brown and Reichard, 1969). In *E. coli* class Ia RNR, this regulation is achieved by changes in the oligomeric arrangement of the α_2_ and β_2_ subunits (Brown and Reichard, 1969; Rofougaran et al., 2008; Ando et al., 2011). When ATP is bound at the activity site, an α_2_β_2_ complex is favored. Although no X-ray structure of the active complex has been determined, low resolution models have been generated using small-angle X-ray scattering (Ando et al., 2011), electron microscopy (Minnihan et al., 2013), and distance measurements made through spectroscopic analyses (Seyedsayamdost et al., 2007) (Figure 1D). This active α_2_β_2_ complex is capable of a long-range proton coupled electron transfer from β_2_ to α_2_, forming a transient thiyl radical on Cys439 to initiate catalysis (Licht et al., 1996). Alternatively, when concentrations of dATP become too high in the cell, dATP binds at the allosteric activity site and formation of an α_4_β_4_ complex is promoted. The structure of this complex was recently solved (Ando et al., 2011), revealing a ring of alternating α_2_ and β_2_ units that cannot form a productive electron transfer path, thus inhibiting the enzyme (Figure 1D).

**Table 1.** Previously determined binding affinities for substrates in the absence and presence of specificity effectors or analogs. **DOI:** http://dx.doi.org/10.7554/eLife.07141.004

Substrate	K_d_ with no specificity effector	K_d_ with effector or effector analog
CDP	1 mM (von Döbeln and Reichard, 1976), 0.3 mM (Crona et al., 2010)	dAMP-PNP: 88 μM (von Döbeln and Reichard, 1976)
UDP	n.d.	K_M_ with ATP: 220 μM (Larsson and Reichard, 1966)
ADP	420 μM (von Döbeln and Reichard, 1976)	dGTPγS: 70 μM (von Döbeln and Reichard, 1976)
GDP	110 μM, 80 μM (von Döbeln and Reichard, 1976; Crona et al., 2010)	TTP: 22 μM (von Döbeln and Reichard, 1976; Crona et al., 2010)

The second form of allosteric regulation is specificity regulation, which maintains the proper relative ratios of dNTPs in the cell. Briefly, the binding of (d)NTP effectors to an allosteric specificity site in α_2_ influences the preference of RNR for its four nucleoside diphosphate (NDP) substrates. Whereas high levels of dATP inhibit class Ia RNR, at lower levels, dATP promotes CDP or UDP reduction. Likewise, TTP promotes GDP reduction, and dGTP promotes ADP reduction (Figure 1E) (Brown and Reichard, 1969; Rofougaran et al., 2008; von Döbeln and Reichard, 1976). Importantly, the affinity of the α_2_ and β_2_ subunits for each other is weak (~0.4 μM) in the absence of effectors, whereas the binding of a complementary substrate/specificity effector pair increases the affinity of the class Ia RNR subunits fivefold (Crona et al., 2010; Hassan et al., 2008). Previous structural work, which includes: X-ray structures of GDP and TTP bound to *E. coli* α_2_ (Eriksson et al., 1997), structures of all four substrate/effector pairs bound to class Ia α_2_ from *Saccharomyces cerevisiae* (Xu et al., 2006), and class II α_2_ from *Thermotoga maritima* (Larsson et al., 2004), revealed the location of the allosteric specificity sites at the ends of a four helix bundle at the dimer interface (Figure 1B). These data, and accompanying in vitro and in vivo studies on *S. cerevisiae* (Ahmad et al., 2012; Kumar et al., 2010; Kumar et al., 2011), also implicated which residues (Gln294 and Arg298, *E. coli* numbering) and which regions of the structure are involved in the communication between the specificity site and the active site. A flexible loop, termed loop 2 (residues 292–301 in *E. coli*) (Figure 1B), bridges the two sites, which are approximately 15 Å apart, and becomes more ordered upon effector and substrate binding (Eriksson et al., 1997). An additional loop, termed loop 1 (residues 259–278), is also near the effector-binding site and is stabilized upon effector binding (Eriksson et al., 1997). Thus, previous work established the players involved in allosteric specificity regulation in RNR. This work supports those assignments, and goes on to provide a novel set of crystallographic snapshots that reveal how these residues in the prototypic RNR from *E. coli* are able to communicate and thereby regulate substrate preference.

## Results

**Figure 2.**
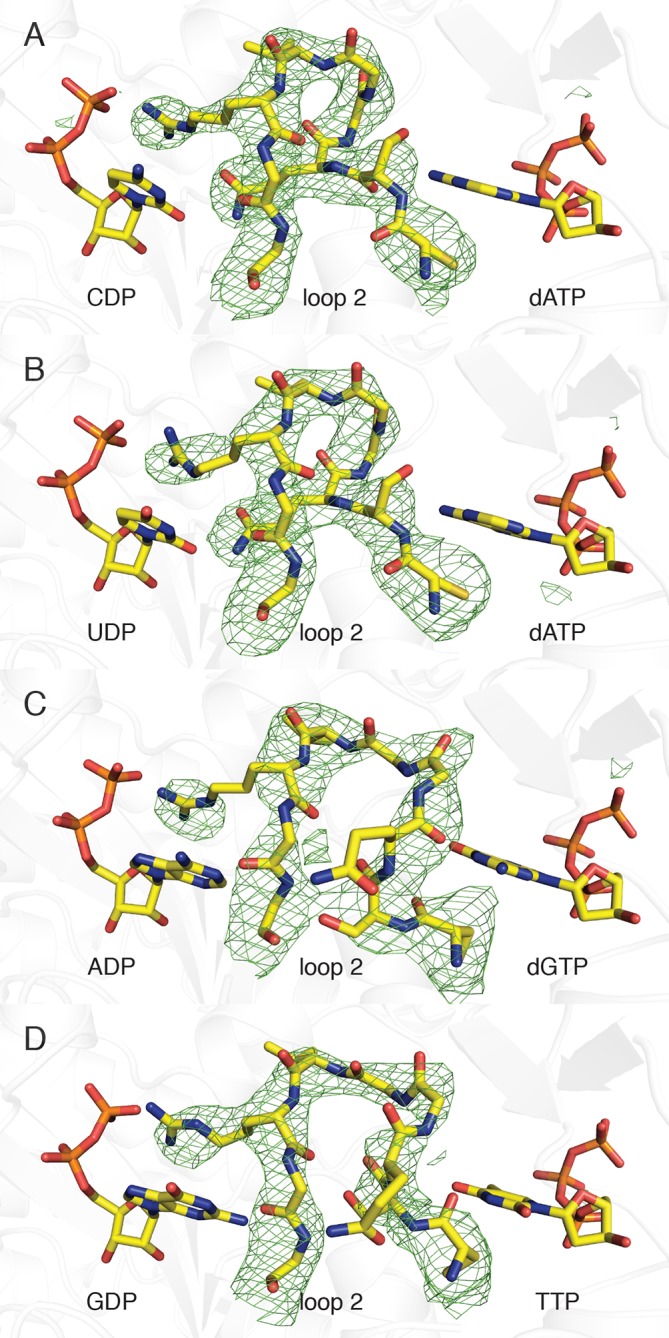
Composite omit electron density confirms that loop 2 is ordered in our *E. coli* class Ia RNR structures. Protein is shown as grey ribbons with substrate, loop 2, and specificity effector in sticks and labeled. Carbon is colored in yellow, oxygen in red, nitrogen in blue, phosphorus in orange. 2*F*_o_−*F*_c_ composite omit density is shown contoured at +1.0 σ (green mesh). (**A**) α_4_β_4_-CDP/dATP structure. (**B**) α_4_β_4_-UDP/dATP structure. (**C**) α_4_β_4_-ADP/dGTP structure. (D) α_4_β_4_-GDP/TTP structure. **DOI:** http://dx.doi.org/10.7554/eLife.07141.005

We have utilized an α_4_β_4_ crystal form of the *E. coli* class Ia RNR (Figure 1D, right) (Ando et al., 2011; Zimanyi et al., 2012) to obtain structures of all four substrate/specificity effector pairs (CDP/dATP, UDP/dATP, ADP/dGTP or GDP/TTP) bound to the α_2_ subunit (Table 2). Each model consists of two α_2_ dimers, two β_2_ dimers, and activity effector dATP in all four allosteric activity sites. The residues modeled for each chain of each structure are given in Table 3. Although the α_4_β_4_ form of the enzyme cannot turn over substrates due to abrogation of the radical transfer pathway, the substrate and effector binding sites are not affected (Video 1). In all structures, substrates and effectors were found ordered at their respective binding sites (loop 2 electron density shown in Figure 2).

**Table 2.** X-ray data collection and refinement statistics. **DOI:** http://dx.doi.org/10.7554/eLife.07141.006

	α_4_β_4_ complex with dATP in allosteric activity site and the following substrate/specificity effector pairs:			
	CDP/dATP	UDP/dATP	ADP/dGTP	GDP/TTP
				
**Data collection**				
Space group	*C*2	*C*2	*C*2	*C*2
Cell dimensions				
a, b, c (Å); β (°)	274.2, 157.8, 164.5; 118.8	274.6, 157.3, 164.2; 119.3	274.3, 157.1,166.0; 119.7	274.8, 157.8, 165.8; 119.5
Wavelength (Å)	0.9792	0.9795	0.9795	0.9795
Resolution (Å) ^a^	50.0 – 2.97 (3.08 – 2.97)	50.0 – 3.25 (3.37 – 3.25)	50.0 – 3.40 (3.52 – 3.40)	50.0 – 3.20 (3.31 – 3.20)
*R*_sym_^a,b^	9.3 (52.7)	9.6 (57.7)	12.7 (49.9)	11.0 (61.9)
<*I*/σ*I*> ^a^	13.9 (2.2)	13.1 (2.3)	8.1 (2.4)	11.8 (2.2)
Completeness (%)^a^	98.9 (99.6)	98.8 (99.2)	93.6 (95.2)	98.8 (99.3)
Redundancy ^a^	3.7 (3.5)	3.9 (3.8)	3.0 (2.9)	3.5 (3.6)
				
**Refinement**				
Resolution (Å)	50.0 – 2.97	50.0 – 3.25	50.0 – 3.40	50.0 – 3.20
Number of reflections	123,362	95,095	78,583	99,942
*R*_work_/*R*_free_ (%)^c^	19.0/21.4	19.2/22.1	18.4/22.1	19.0/21.9
Number atoms/molecules				
Protein atoms	34919	34898	34905	34993
Fe^2+^/Mg^2+^ atoms	8/8	8/8	8/8	8/8
Activity site nucleotides	4	8	4	8
Substrate/effector pairs	4/4	4/4	4/4	4/4
Water molecules	143	59	94	61
Average *B*-factors (Å^2^)				
Protein	57.4	80.3	67.0	70.9
Fe^2+^/Mg^2+^	45.5/73.2	66.7/90.2	57.7/75.3	59.0/80.4
Activity site nucleotides	71.5	101.1	81.9	87.3
Substrate/Effector pairs	53.4/63.0	78.4/77.5	62.4/64.0	66.0/68.6
Loop 2	56.3	82.9	65.6	74.1
Water	52.5	68.3	61.2	62.4
Root-mean-square deviations				
Bond lengths (Å)	0.003	0.003	0.004	0.004
Bond angles (°)	0.633	0.651	0.667	0.677
Ramachandran plot (%)				
Favored	93.6	92.1	93.2	93.0
Allowed	6.1	7.6	6.4	6.7
Generously allowed	0.2	0.3	0.3	0.3
Disallowed	0.1	0.0	0.1	0.0
^a^Value in parentheses is for highest resolution bin.				
^b^$R_{sym}=\left(\sum_{hkl}\sum_{i}\left|I_{i}(hkl)\;-\;<I(hkl)>\right|\right)/\left(\sum_{hkl}\sum_{i}I_{i}\left(hkl\right)\right)$.				
^c^*R*_free_ was calculated from 5% of the total reflections and was maintained across all four data sets.

**Table 3.** Residues modeled for each chain of α (1–761) and β (1–375) in all four α_4_β_4_ structures. In all four structures, the following regions are disordered and cannot be seen in the experimental electron density: the last ~24 C-terminal residues of α that contain redox active cysteines Cys754 and Cys759 and the ~20 residues of β that connect residue 330 to the ~15 C-terminal residues (360–375) that bind to the α subunit. **DOI:** http://dx.doi.org/10.7554/eLife.07141.007

	Chain							
Structure	A (α)	B (α)	C (α)	D (α)	E (β)	F (β)	G (β)	H (β)
CDP/dATP	5-736	4-737	5-736	4-737	1-339, 363-375	1-341, 360-375	1-341, 360-375	1-340, 361-375
UDP/dATP	4-737	4-737	4-737	4-736	1-339, 363-373	1-341, 360-375	1-341, 360-375	1-340, 361-375
ADP/dGTP	5-736	4-736	4-736	4-736	1-339, 363-375	1-341, 360-375	1-341, 360-375	1-340, 361-375
GDP/TTP	1-736	5-736	4-737	4-737	1-339, 363-375	1-341, 360-375	1-341, 360-375	1-344, 361-375

**Video 1.** Location of active site and specificity site in α_4_β_4_ complex of *E. coli* class Ia RNR. **DOI:** http://dx.doi.org/10.7554/eLife.07141.008

### Substrate/effector binding induces a clamping of the (β/α)_10_ barrel in the α_4_β_4_ crystal form

**Figure 3.**
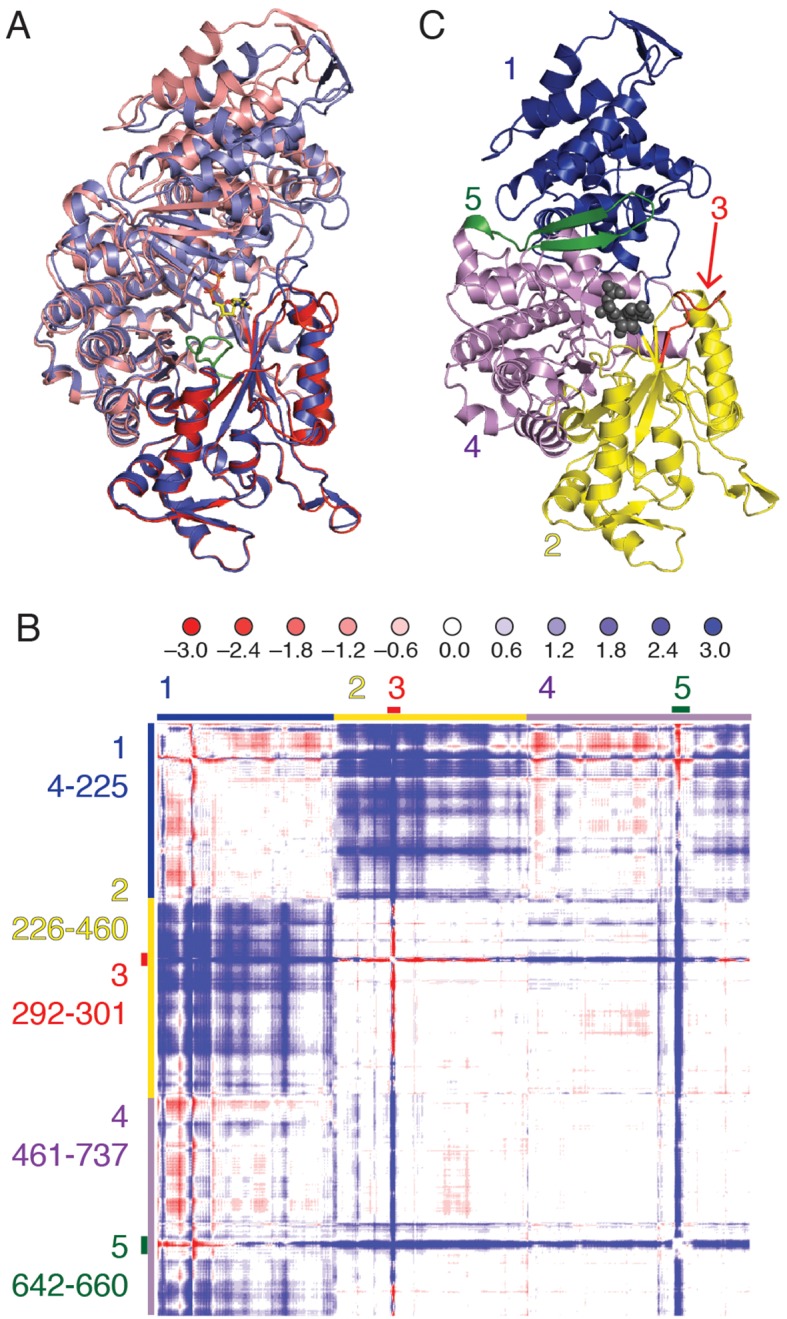
Cα difference distance matrix plot reveals movements that occur concurrent with substrate binding for *E. coli* class Ia RNR. (**A**) Superposition of α from our CDP/dATP structure (N-terminus and one half of the active site barrel in purple and the other half barrel in blue) and α from a substrate-free *E. coli* RNR structure (PDB ID: 3R1R, (Eriksson et al., 1997)) (N-terminus and one half of active site barrel in pink and the other half barrel in red). The two chains were aligned by the active site finger loop, which is colored green. CDP is shown as sticks. (**B**) Distances in chain A of the CDP/dATP structure (this work) were subtracted from chain A of the substrate-free α_2_ structure (PDB ID: 3R1R) for residues 4–737. Scale is shown on the top and is ±3.0 Å (positive values in blue indicate a shorter distance in the CDP/dATP structure and negative values in red indicate a longer distance). Regions that move in a concerted fashion are indicated with colored lines and residue ranges are listed to the left of the plot. (**C**) One α chain is shown in ribbons with residue ranges from (**B**) colored. Region 1 (blue), the N-terminal 225 residues, contracts towards region 2 (yellow). Region 3 (red) includes loop 2 residues and moves towards the active site (in region 4). A flexible loop, region 5 (green), undergoes a large motion towards regions 2 and 4 whereas region 4 undergoes little movement with respect to the rest of the structure. **DOI:** http://dx.doi.org/10.7554/eLife.07141.009

Each substrate/effector bound α_4_β_4_ structure reveals a closing of the (β/α)_10_ barrel around the bound substrate that is not observed in the absence of substrate. An initial superposition of one α chain from the previously reported free α_2_ soaked with AMP-PNP (PDB ID: 3R1R (Eriksson et al., 1997)) onto the α_4_β_4_ crystal form was performed using only residues 432–446, that comprise the conserved active site loop (‘finger loop’) found in the center of the (β/α)_10_ barrel. In this structural alignment, one half of the active site barrel overlays exactly, whereas the second half of the barrel and the N-terminal portion of the structure has undergone a clamping movement (Figure 3). To better characterize this movement, a difference distance matrix (DDM) was calculated. This plot reveals relative movements in a reference-independent manner. Briefly, the distance between every possible pair of atoms in one chain of α from the α_4_β_4_ structure is subtracted from the same distance measured in the free α_2_ structure, giving a representation of how much the distances between pairs of atoms change between the two structures. The pattern of movements seen in the plot indicates that the N-terminal 220 residues (region 1 of Figure 3B,C) shift substantially (>3Å), bringing them closer to half of the active site barrel (residues ~224–439, region 2 of Figure 3B,C). We also observe that loop 2 (region 3 of Figure 3B,C) and a flexible β hairpin that sits adjacent to the N-terminus (residues 646–651, region 5 of Figure 3B,C) undergo substantial motions.

**Figure 4.**
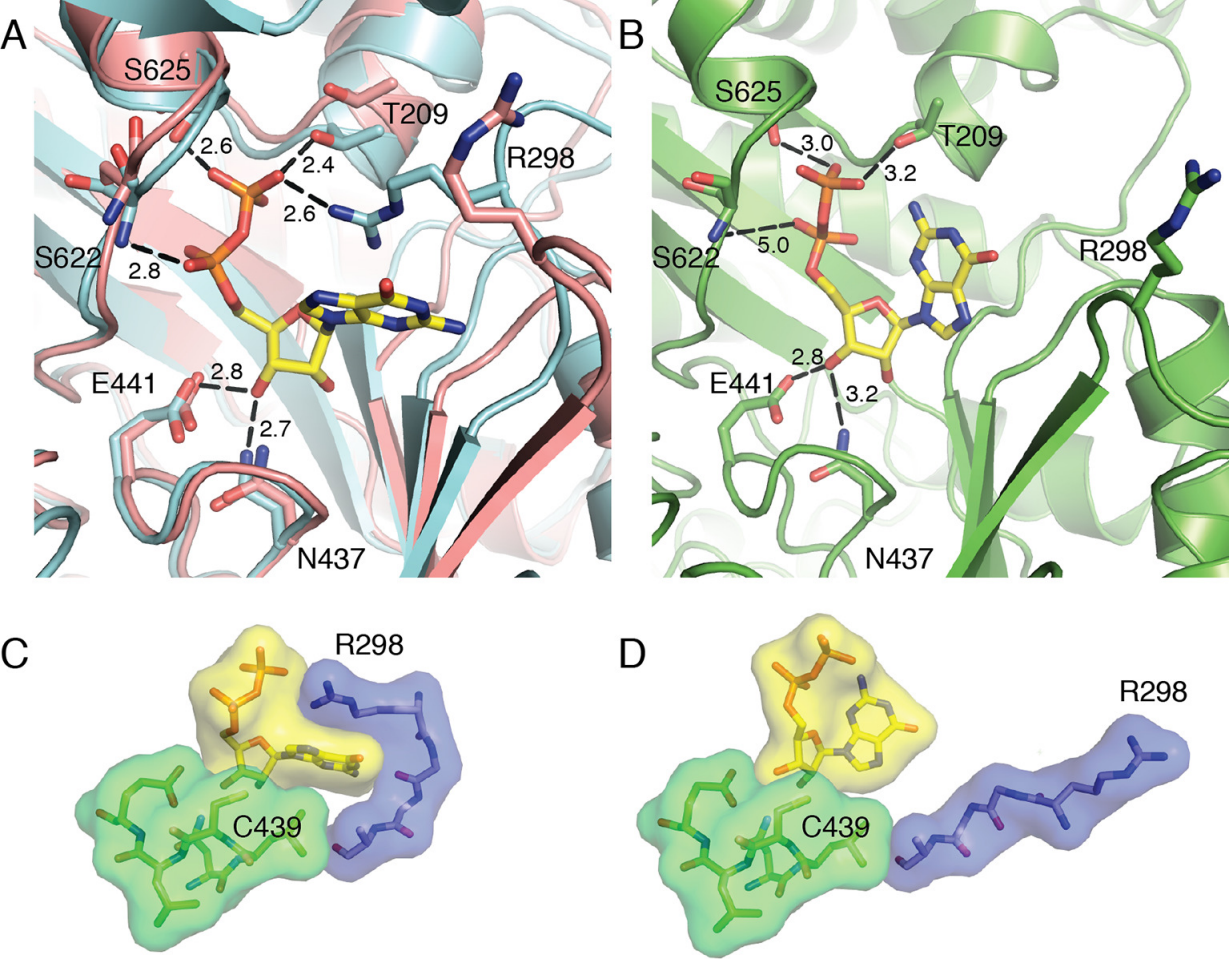
Local movements stabilize substrate binding to the active site of α_2_. (**A**) GDP/TTP-bound structure (this work, cyan) is overlaid with substrate free α_2_ (PDB ID: 3R1R, pink). Distances are given in Å. GDP and residues that form hydrogen bonds to GDP are shown as sticks with GDP carbons in yellow, protein carbons in cyan or pink. Ser622, Ser625 and Thr209 move to form hydrogen bonds to the phosphates of GDP. Arg298 of loop 2 reaches over the guanine base to contact the phosphates. (**B**) Previously reported α_2_ structure with GDP bound at an occupancy of 0.5 (PDB ID: 4R1R). GDP and residues that form hydrogen bonds to GDP are shown in sticks with GDP carbons in yellow and protein carbons in green. (**C**) Van der Waals packing around GDP in GDP/TTP structure (this work) showing a tightly packed active site. (**D**) Van der Waals packing around GDP from previously reported α_2_ structure (PDB ID: 4R1R) showing that the active site is still open. **DOI:** http://dx.doi.org/10.7554/eLife.07141.010

**Figure 5.**
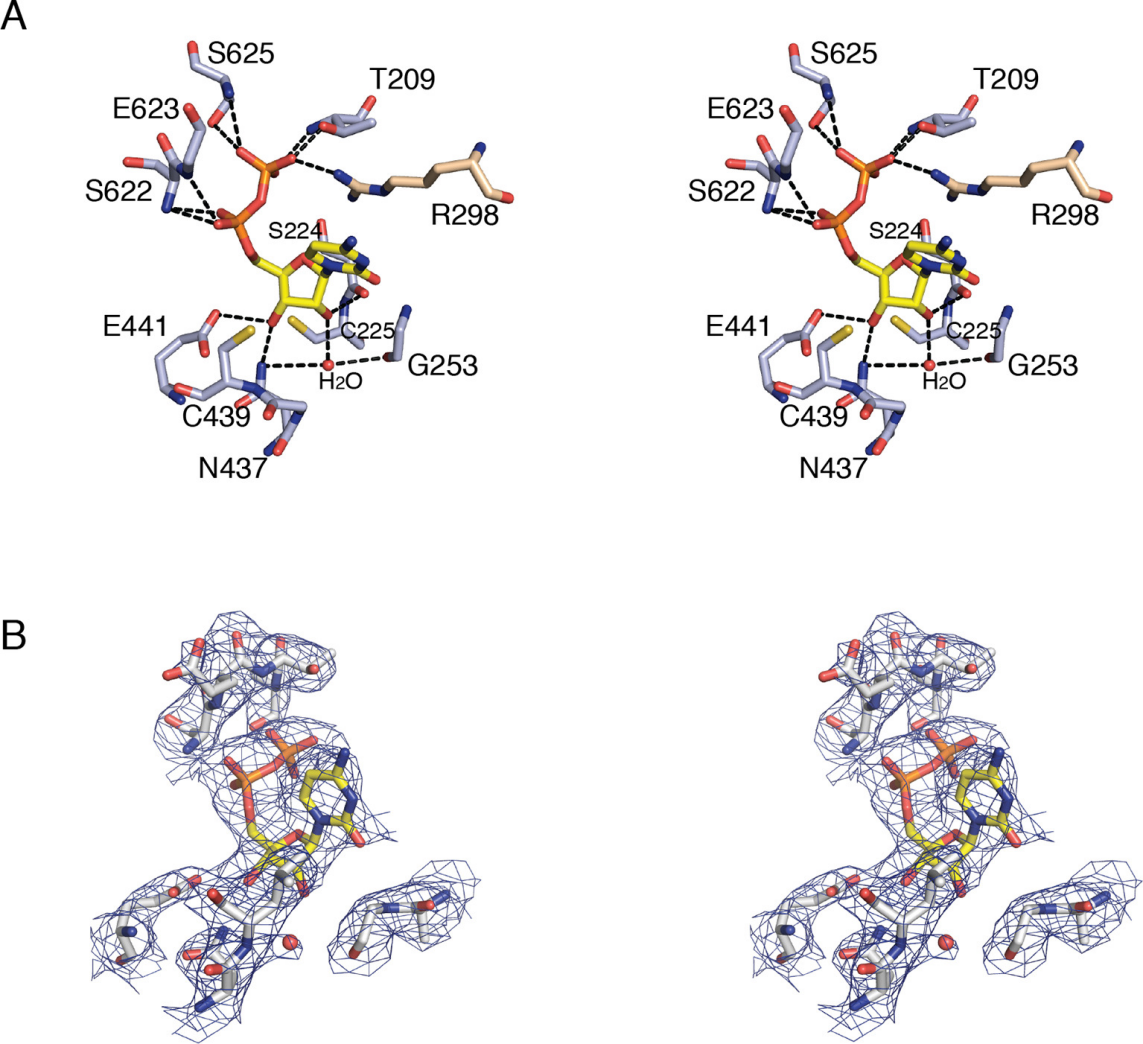
Details of CDP binding to a clamped-down active site in *E. coli* class Ia RNR. (**A**) Wall-eyed stereo view of CDP ribose and phosphate interactions with protein. CDP is shown as sticks with carbons in yellow. Protein side chain carbons are colored light purple and loop 2 residue, Arg298, carbons are colored tan. A putative water molecule is shown as a red sphere. Hydrogen-bonding interactions, shown with black dashed lines, include: O3' of ribose to Glu441, the proposed general base (van der Donk et al., 1996; Persson et al., 1997), and O2' to backbone carbonyl of Ser224. Cys225 is the proposed proton donor for the 2'-OH that is lost as H_2_O. Cys225 is 3.4–3.6 Å from the O2'. The distance between the sulfur atom of Cys439, where the thiyl radical is formed, and C3' of the ribose, where a hydrogen atom is abstracted to initiate catalysis, is 3.5–3.7 Å. (**B**) Wall-eyed stereo view of omit electron density for CDP structure shown in A. Orientation is tilted and rotated slightly to show the water density. A water molecule is present in this position in some substrate-bound RNR structures and not others, the significance of which is not clear. Arg298 is not shown for simplicity. **DOI:** http://dx.doi.org/10.7554/eLife.07141.011

Closer inspection of the active site in the superposition (again, aligned by the active site finger loop) reveals that the movements described above directly affect substrate binding (Figure 4A). Residues Ser622, Ser625, and Thr209 form hydrogen bonds with the NDP substrate, whereas in the substrate-free state (pink ribbon in Figure 4A), these residues are shifted away from the center of the barrel. Thr209 is part of the N-terminal region that is shown to move in the DDM analysis. The structure superposition and DDM analysis also indicate that loop 2 shifts, bringing Arg298 closer to the active site. The Arg298 side chain now forms a cation-π interaction with the substrate base and a charge-charge interaction with the substrate β-phosphate. In the substrate-free structure, this side chain is pointing in the opposite direction. Protein contacts like Arg298 to the substrate diphosphate are expected to be particularly important for charge neutralization in enzymes, such as *E. coli* RNR, that do not employ metal cations such as Mg^2+^ for this purpose. The positions of residues that contact the ribose O3' (Glu441 and Asn437) are virtually unchanged between the NDP-free and NDP-bound structures (Figure 4A). All four of our substrate-bound structures show almost identical contacts between the enzyme and the substrate ribose and phosphates as described here and shown in Figure 4A,C and Figure 5 for GDP and CDP, respectively.

In contrast to the compact α_2_ barrel observed in our substrate/effector-bound α_4_β_4_ structures, no clamping motion of the barrel is observed between substrate-free α_2_ and a previously reported structure of α_2_ with GDP substrate and TTP effector bound (Eriksson et al., 1997). In this structure, Ser622, Ser625, and Thr209 are all 3.0 Å or greater from the substrate diphosphate, and Arg298 points away from the active site (Figure 4B). These weaker (or missing) interactions between protein and substrate in this previously determined structure are consistent with the fact that substrate is observed in an altered orientation in the active site (Figure 4C,D) and is only present at half occupancy. We attribute the differences in substrate positioning in this structure to the inability of the active site barrel to clamp around substrate due to crystal lattice contacts. Symmetry-related molecules are closer to the active site in the α_2_-only crystalline state than they are in the α_4_β_4_ crystal form. Importantly, differences in flexibility cannot be attributed to the fact that α_4_β_4_ is an inactive state of *E. coli* RNR because α_2_ is also an inactive state; the former because β_2_ is held at arm’s length from α_2_, and the latter because β_2_ is absent altogether. Additionally, other than contributing to an amenable crystal lattice, there is nothing to suggest that β_2_ must be present for α_2_ to clamp. When lattice contacts do not restrain α_2_ movement, α_2_ should be able to clamp in the presence of a cognate substrate/effector pair regardless of β_2._ Thus, it seems that different crystal forms have given us two distinct snapshots of substrate-bound states for the *E. coli* class Ia enzyme: a high affinity state which is closed and ready for radical-based chemistry when β_2_ becomes available (Figure 4C) and a lower affinity state in which substrate is still exposed to solvent and not ready to undergo catalysis, regardless of the availability of β_2_ (Figure 4D).

### Specificity effectors are anchored through conserved ribose and phosphate interactions

**Figure 6.**
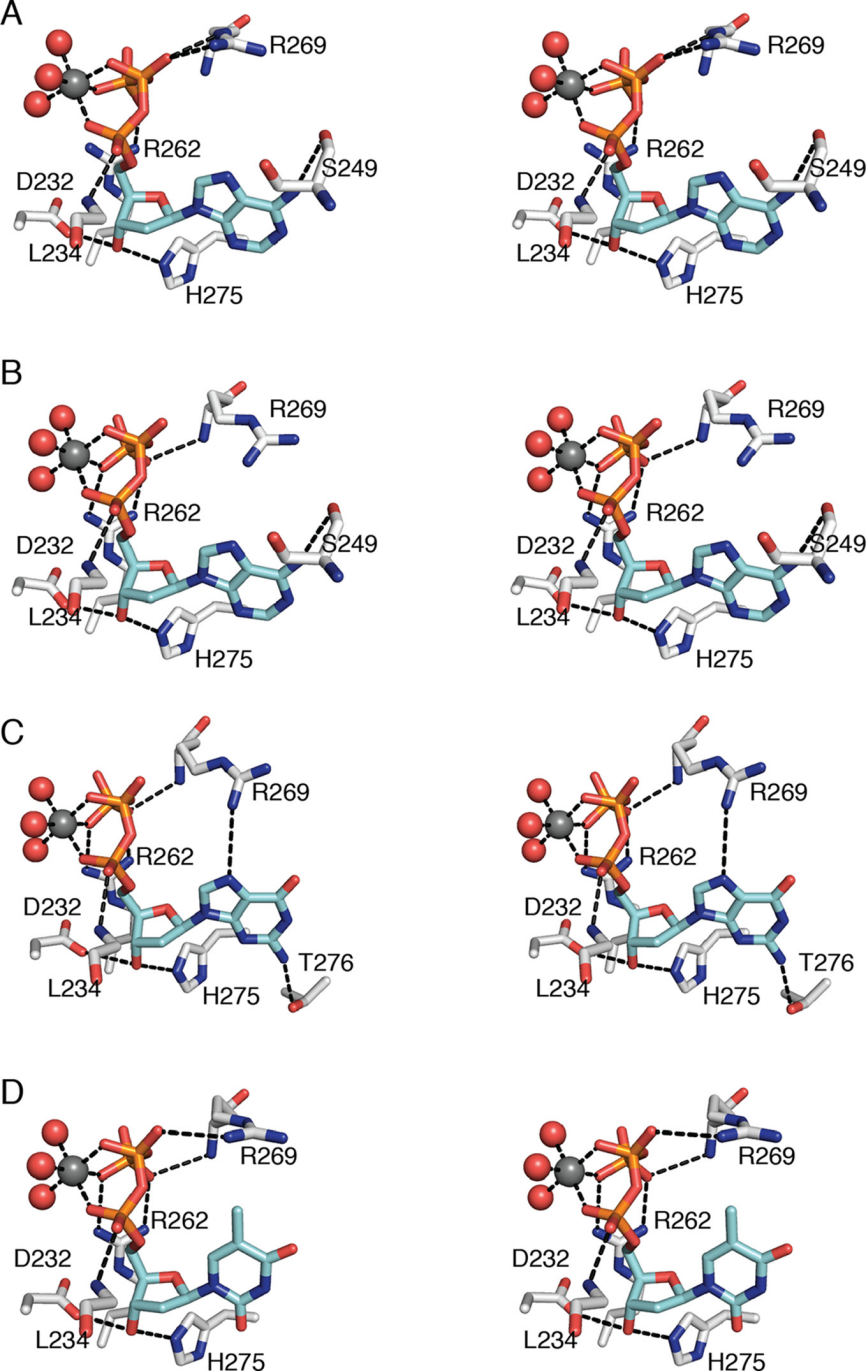
Interactions that anchor specificity effectors in *E. coli* class Ia RNR involve residues outside of loop 2. Interactions are shown for (**A**) CDP/dATP, (**B**) UDP/dATP, (**C**) ADP/dGTP, and (**D**) GDP/TTP. The dNTP effector carbons are colored cyan and the protein carbons are colored grey. Magnesium ions are colored grey and waters in red. The side chain of Leu234 is omitted for clarity. Hydrogen-bonding interactions are shown with black dashed lines. Figures are displayed in wall-eyed stereo. **DOI:** http://dx.doi.org/10.7554/eLife.07141.012

Regardless of the identity of the dNTP base, the ribose and phosphates of the three specificity effectors (dATP, dGTP, and TTP) form almost identical contacts with RNR (Figure 6). Despite the presence of the specificity effector site at the dimer interface, these common interactions are made with only one chain of the α_2_ dimer. Asp232 and His275 are within hydrogen-bonding distance of O3' of the ribose. One Mg^2+^ ion appears to form an octahedral coordination complex with three phosphate oxygens of the specificity effector and three water molecules (Figure 6). There are also hydrogen bonds between the phosphates and the backbone amides of residues Arg269 and Leu234 and charge-charge interactions with the side chains of Arg269 and/or Arg262.

### Three different loop 2 conformations are responsible for substrate specificity

**Figure 7.**
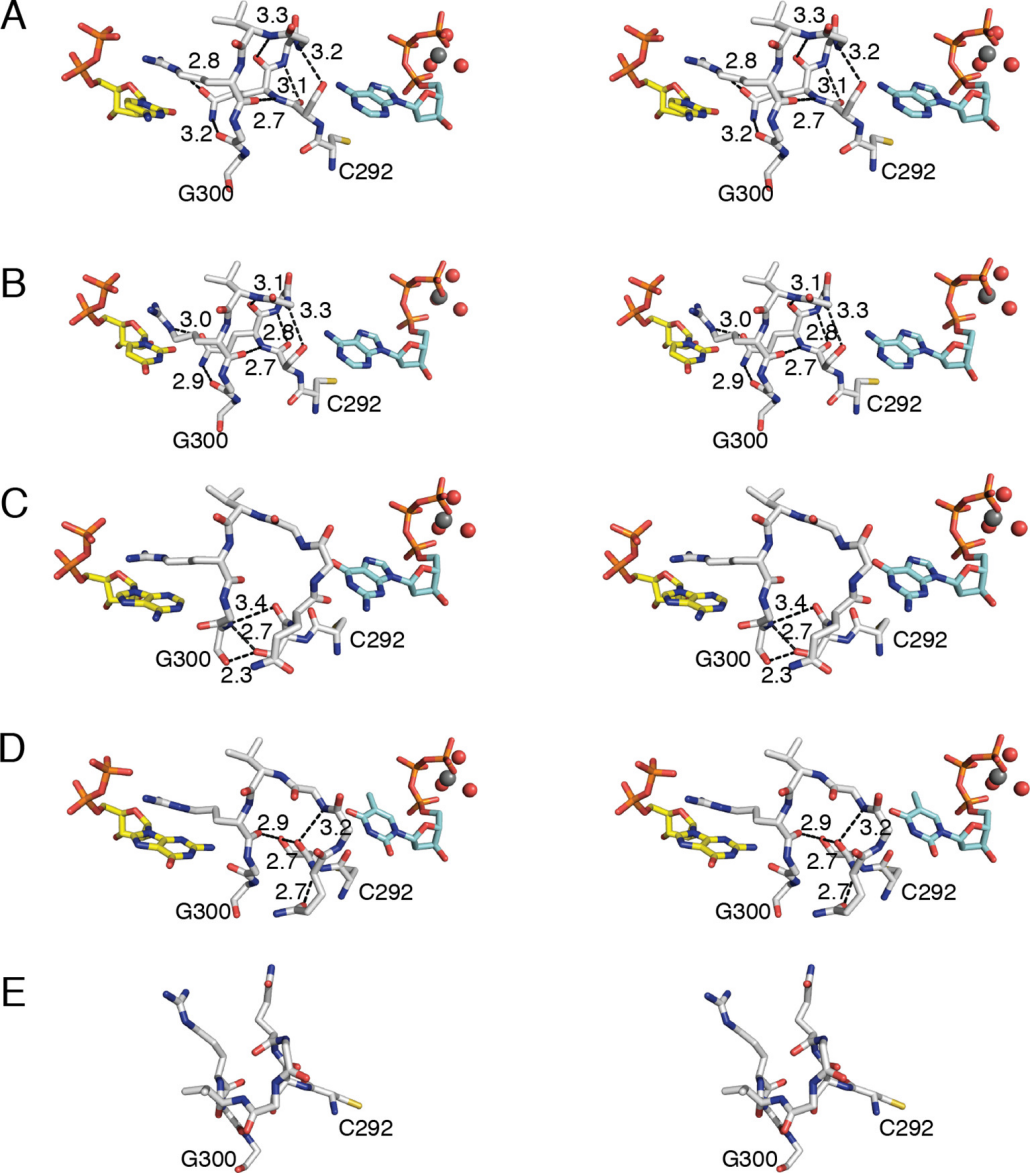
Conformations of *E. coli* class Ia RNR loop 2 in the presence and absence of substrate-effector pairs. Structures are shown in wall-eyed stereo as sticks for (**A**) CDP/dATP, (**B**) UDP/dATP, (**C**) ADP/dGTP, and (**D**) GDP/TTP. (**E**) α_2_ with no substrates or effectors bound (PDB ID: 3R1R). Substrates are shown with carbons in yellow, effectors are shown with carbons in cyan, and loop 2 is shown with carbons in grey. Other atoms colored as in previous figures. Hydrogen-bonding interactions are shown in black dashed lines with distances given in Å. Only interactions between protein residues are shown. **DOI:** http://dx.doi.org/10.7554/eLife.07141.013

**Figure 8.**
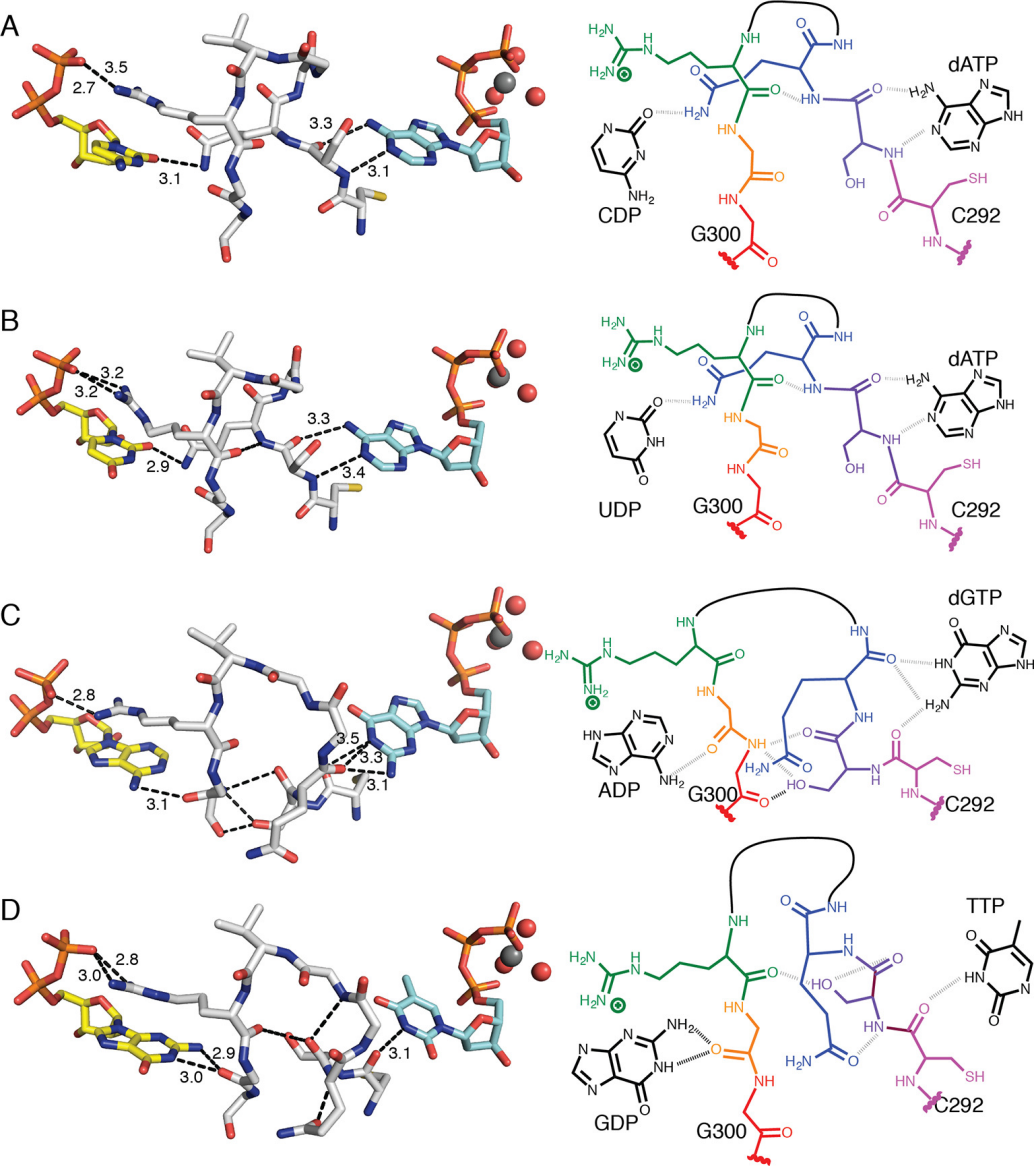
Molecular basis for communication between substrate and effector binding sites in *E. coli* class Ia RNR. Structures are shown as sticks on the left with 2D representation of hydrogen-bonding interactions on the right for (**A**) CDP/dATP, (**B**) UDP/dATP, (**C**) ADP/dGTP, and (**D**) GDP/TTP. Atoms are colored as in Figure 7. Hydrogen-bonding interactions are shown in black dashed lines with distances given in Å. In the 2D representation, each residue is colored a different color, residues 295–297 are shown as a black line, and hydrogen bonds are indicated with black dashed lines. **DOI:** http://dx.doi.org/10.7554/eLife.07141.014

Loop 2 is ordered in all four structures as indicated by 2*F*_o_–*F*_c_ composite omit electron density (Figure 2). As noted above, Arg298 of loop 2 forms a conserved interaction with the β-phosphate of all four substrates (Figures 7, 8). On the effector side of loop 2, Cys292 packs against the base of all three effectors (Figures 7, 8), consistent with binding studies that show the affinity for all specificity effectors is decreased when Cys292 is mutated to alanine (Ormö and Sjöberg, 1996). The remaining interactions with loop 2 are distinct for each substrate/effector pair (Figures 7, 8). dATP, which preferentially increases RNR activity for CDP and UDP substrates, makes two hydrogen bonds with loop 2. The backbone amide and carbonyl of Ser293 are in position to hydrogen bond to N1 and N6, respectively, of the adenine base of dATP. These hydrogen bonds hold Ser293 in place and position the adjacent Gln294 in to a conformation that points into the substrate-binding site (Figure 8A,B). In this conformation, Gln294 can form a hydrogen bond with O2 of CDP or UDP. There is no difference in substrate positioning between CDP and UDP, as the unique positions of the pyrimidine ring (N2 and N4/O4) are not involved in any contacts with the protein. Furthermore, the positioning of Gln294 by dATP selects for pyrimidine binding, as binding of a purine base would be disfavored due to steric occlusion by Gln294.

dGTP, which preferentially increases RNR activity for substrate ADP, forms one hydrogen bond with loop 1 residue Thr276 (Figure 6C) in addition to its loop 2 contacts. It is the only effector for which a hydrogen bond is formed between the base and loop 1. Because the position of hydrogen bond donors and acceptors at N1 and O6 in dGTP is opposite from that of N1 and N6 of dATP, the interaction with the backbone amide of Ser293 in loop 2 is unfavorable in this structure. Instead, Ser293 is shifted away from the base, and N1 and N2 – both hydrogen bond donors – form an interaction with the backbone carbonyl of Gln294, which has shifted away from the substrate (Figure 7A–C and 8A–C). Ser293 thus makes no hydrogen bond contacts to dGTP and instead provides hydrogen bonds to Gly300 on the other side of loop 2 through both its side chain and backbone. Stabilization of Gly300 in this position in turn allows for a hydrogen bond between the carbonyl of Gly299 with the N6 position of the preferred substrate, ADP. The movement of Gln294 away from the substrate-binding site allows room for the larger ADP to bind. Thus, Ser293 and Gln294 are responsible both for differentiating between the purine bases adenine and guanine at the effector-binding site and for differentiating pyrimidines from purines at the substrate site, either directly (in the case of Gln294 hydrogen bonding to CDP or UDP) or indirectly through stabilization of Gly299 and Gly300. With dATP bound, Ser293 plays a role in effector recognition and Gln294 in substrate recognition; these roles are reversed for ADP/dGTP.

TTP, which preferentially increases RNR activity for substrate GDP, binds to the specificity site such that only the carbonyl of Cys292 from loop 2 is positioned to form a hydrogen-bonding interaction with the thymine base (Figure 8D). As in the ADP/dGTP structure, Gln294 is swung away from the active site to make room for the binding of a purine substrate (Figure 8D). On the substrate side of loop 2, N1 and N2 of GDP are both within hydrogen-bonding distance of the backbone carbonyl of Gly299. N2 of GDP is also within hydrogen-bonding distance of the backbone carbonyl of Ala252 (not shown), giving GDP one more hydrogen bond than the other substrates

For the purine substrates ADP and GDP, preference appears to rely on a shift of the carbonyl of Gly299 in loop 2 forward or backward in the active site, enabled by the binding of dGTP or TTP, respectively, at the specificity site. With dGTP bound, Ser293 is positioned such that it pushes the carbonyl of Gly299 'forward' (away from the effector-side of loop 2, see Figure 8C), allowing a hydrogen bond to form between the carbonyl of Gly299 and N6 of adenine. With the smaller TTP in the effector site, Ser293 rearranges such that the carbonyl of Gly299 can relax ‘backward’ (toward the effector-side of loop 2, see Figure 8D), allowing for a hydrogen bond between the carbonyl of Gly299 and N1 and N2 of guanine. Specificity is imparted by the fact that the ‘forward’ position of the Gly299 carbonyl interacts with the 6-position of the purine, which is a hydrogen bond donor (NH_2_) in adenine and a hydrogen bond acceptor (O) in guanine; and the ‘backward’ position of the Gly299 carbonyl interacts with the 1-position and 2-position of the purine, which are both hydrogen bond donors (NH and NH_2_) in guanine but not in adenine (Figure 8C,D). To briefly summarize purine specificity, purines are favored over pyrimidines when Gln294 is positioned away from the active site creating a larger substrate-binding pocket, and ADP is favored over GDP when dGTP binding stabilizes Ser293 close to the Gly299 backbone pushing its carbonyl forward, and GDP is favored over ADP when TTP allows Ser293 to fall back.

### Activity of mutant proteins Gln294Ala-α_2_ and Arg298Ala-α_2_ support structural observations

**Figure 9.**
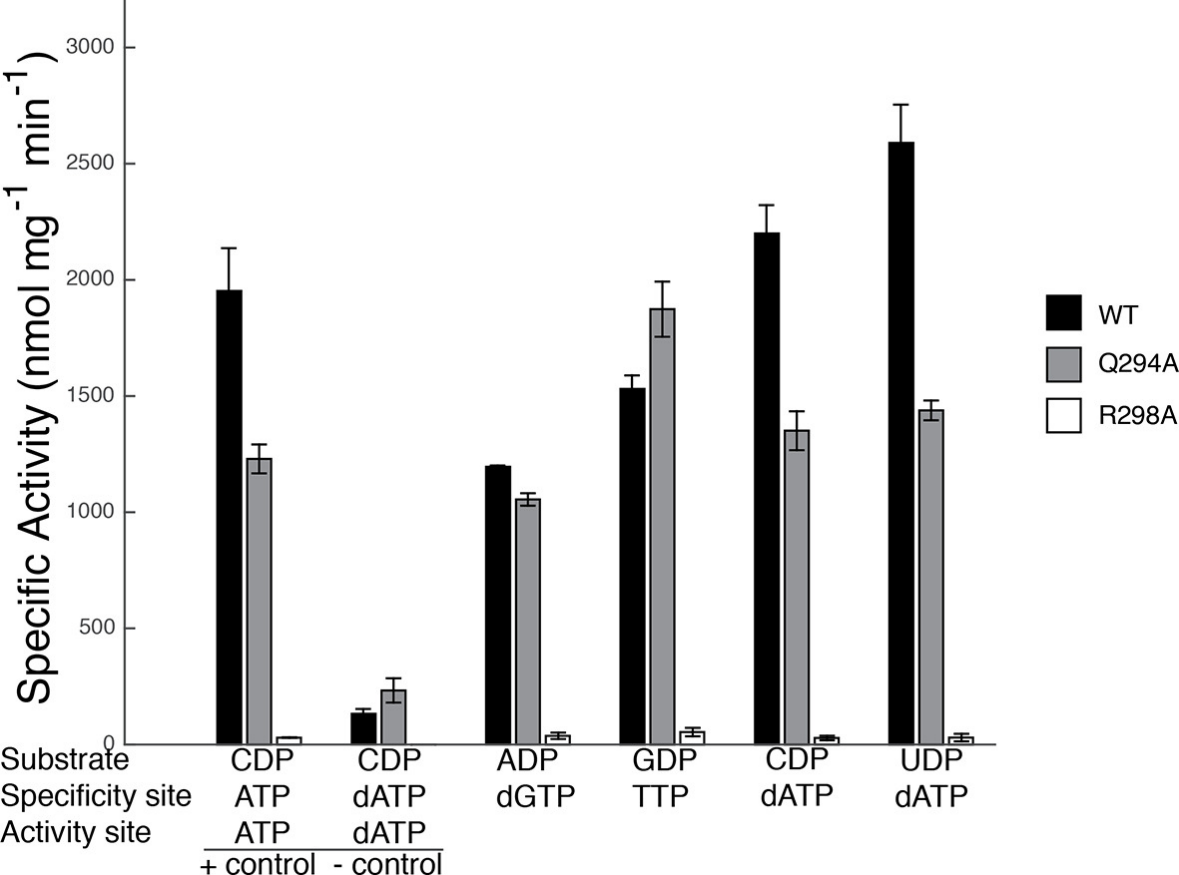
Specific activity for wild-type and mutant forms of *E. coli* RNR in the presence of different substrate/effector pairs. Wild-type is shown in black, Gln294Ala in grey, and Arg298Ala in white. Activity was measured by a coupled assay that follows nicotinamide adenine dinucleotide phosphate (NAPDH ) consumption (see Materials and methods) for the following substrate and effector concentrations: 1 mM CDP and 3 mM ATP (far left), 1 mM CDP and 175 μM dATP (second to left), 1 mM ADP and 120 μM dGTP, 1 mM GDP and 250 μM TTP, and 1 mM CDP/UDP and 1 μM dATP (far right). Since dATP at high concentrations (175 μM) inhibits the enzyme, the sets of bars at the far left represent control experiments to show activity levels under active (CDP/ATP) and inactive (CDP/dATP) conditions. **DOI:** http://dx.doi.org/10.7554/eLife.07141.015

Our sets of structures implicate Arg298 and Gln294 as key residues in the specificity regulation of *E. coli* RNR. To confirm this prediction, we prepared Gln294Ala and Arg298Ala mutant enzymes and tested the activity for each substrate/effector pair. Because dATP at high concentrations inhibits RNR activity by binding to the allosteric activity site, whereas at lower concentrations it is a specificity effector, we first carried out control experiments to determine activity levels for CDP reduction in the absence of dATP and at an inhibitory dATP concentration (175 μM) (Figure 9). Results show that wild-type RNR is as active (CDP/dATP) or more active (UDP/dATP) at 1 μM dATP than it is in the positive control (CDP/ATP), indicating that at 1 μM, dATP is acting as a specificity effector and not as an allosteric inhibitor of activity, consistent with previous studies (Birgander et al., 2004). The negative control shows the substantial decrease in wild-type RNR activity on CDP when the concentration of dATP is high enough (175 μM) for dATP to bind to the allosteric activity site and inhibit the enzyme. With these controls in place, the activity of mutant RNRs with all four substrate/effector pairs can be established. Our structures predict that mutation of Arg298 to alanine should decrease activity for all substrates, and that is exactly what we observe (Figure 9). The effect is dramatic for this mutant, with negligible activity observed in all cases. In fact, the small amount of activity detected could be due to low levels of contamination of wild-type *E. coli* RNR instead of the Arg298Ala mutant protein. Thus, Arg298Ala-RNR is either mostly or completely inactive. In contrast, for Gln294Ala-RNR, we would expect purine and pyrimidine substrates to be differentially affected, with minimal or no loss of activity on ADP/GDP and measurable loss on CDP/UDP, and that is again what we observe. For Gln294Ala, activity is the same as wild-type for ADP/dGTP and perhaps even higher than wild-type for GDP/TTP, whereas for both CDP and UDP with 1 μM dATP, activity is decreased (Figure 9). These results are consistent with the removal of the Gln294 side chain from the active site to create room for the larger substrates (ADP/GDP) to bind and also with the positioning of Gln294 into the active site to stabilize CDP/UDP binding through hydrogen-bonding to the respective bases.

## Discussion

In this work, we have sought to decipher the molecular rules of substrate/effector recognition in RNR and determine the basis for increased substrate affinity in the presence of a cognate effector. We observe that in the absence of substrates or effectors (for example, in the previously reported structure of α_2_ (PDB ID: 3R1R (Eriksson et al., 1997)), the barrel is not clamped and loop 2 is not found in any of the conformations seen in our effector-bound structures (Figure 7E). However, in the presence of substrate/effector pairs, we find a clamped down barrel and a stabilized loop 2 that adopts three different conformations depending on the allosteric effector that is bound (Figure 7A–D) (Video 2). Loop 2 residues Cys292, Ser293, and Gln294 appear to be involved in specific interactions that read out the identity of the base and communicate that identity to the active site. Briefly, dATP, which enhances reduction of CDP/UDP (von Döbeln and Reichard, 1976), makes specific contacts with Ser293, orienting Gln294 toward the active site and stabilizing the binding of both CDP and UDP (Figure 8A–B). The lack of discrimination between CDP and UDP substrates by RNR is not problematic for the cell as cytidine deaminase provides another level of control for dCTP/TTP ratios (Wang and Weiss, 1992; O'Donovan et al., 1971). In contrast, dGTP binding stabilizes Gln294 away from the RNR active site, so that CDP/UDP binding is not stabilized (although it is not prohibited) and there is room for the larger purine substrates to bind and to hydrogen bond to the carbonyl of Gly299 (Figure 8C–D). The creation of a more expansive active site is consistent with the ability of RNR to reduce both purine substrates in the presence of dGTPγS, albeit with a much greater fold activity increase for preferred substrate ADP (von Döbeln and Reichard, 1976). Specificity for ADP versus GDP appears to be modulated by whether effector-loop contacts stabilize the carbonyl of Gly299 in a forward or a backward position, respectively (Figure 8C–D). Thus, our structural data suggest that movement of Gln294 in and out of the active site alternatively shrinks and expands the active site for CDP/UDP versus ADP/GDP, whereas a more modest shift of the Gly299 carbonyl is involved in ADP/GDP selectivity. Mutagenesis of Gln294 to Ala is consistent with this proposal, showing decreased activity on CDP/UDP and no change or increased activity on ADP/GDP (Figure 9).

**Video 2.** Loop 2 movements responsible for allosteric specificity regulation in *E. coli* class Ia RNR. **DOI:** http://dx.doi.org/10.7554/eLife.07141.016

**Figure 10.**
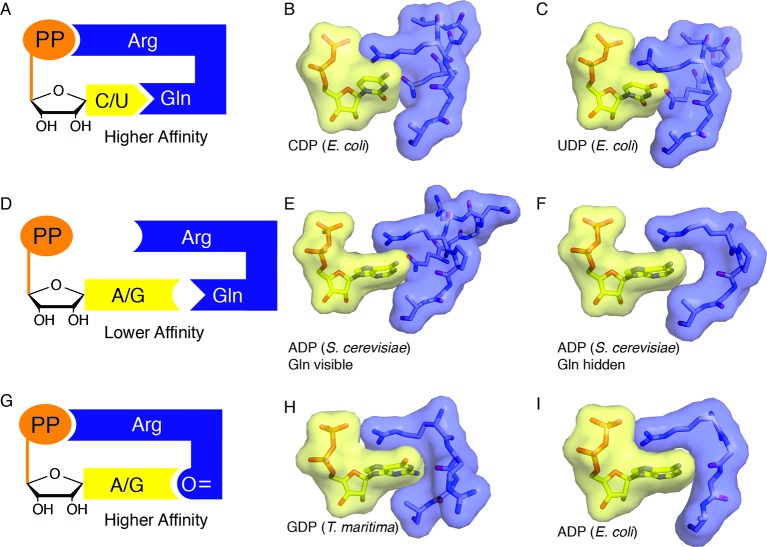
Snapshots of higher and lower affinity substrate-bound states of RNR. (**A**) Cartoon of a high-affinity complex for CDP/UDP bound to RNR. (**B**) Packing of active site in *E. coli* class Ia RNR CDP/dATP structure (this work). (**C**) Packing of active site for UDP/dATP structure (this work). (**D**) Cartoon of a lower-affinity complex in which positioning of Gln into the active site holds loop 2 away such that Arg cannot reach the substrate diphosphate. (**E**) Packing of active site of ADP-bound *S. cerevisiae* RNR structure (PDB ID: 2CVX). With Gln288 (Gln294 in *E. coli*) in the active site, Arg293 (Arg298 in *E. coli*) does not reach the diphosphate of substrate. (**F**) Same structure as in (**E**), but Gln is not shown. Shape complementary suggests that a tighter complex could form than the one that is visualized in this crystal structure. (**G**) Cartoon of a high-affinity complex for ADP/GDP bound to RNR. (**H**) Packing of active site in GDP structure of class II RNR from *T. maritima* (PDB ID: IXJE) is similar to that of the *E. coli* class Ia RNR with ADP/dGTP bound (shown in panel I) and the *E. coli* GDP/TTP structure that is shown in Figure 4C. (**I**) Packing of active site in *E. coli* class Ia RNR with ADP/dGTP bound (this work). **DOI:** http://dx.doi.org/10.7554/eLife.07141.017

When a cognate substrate/effector pair is bound and the barrel is clamped, Arg298 is able to reach across the active site, stack against the NDP base, and hydrogen bond to the NDP β-phosphate, thereby sequestering the substrate in the active site, and neutralizing the substrate negative charge. These interactions yield what appears to be a high affinity substrate-bound state that is sequestered by solvent and thus amenable to radical-based chemistry. To invoke an analogy, Arg298 is like a latch on a suitcase, locking the active site barrel in cases in which the active site is appropriately packed (Figure 10A). When a suitcase is packed with too many clothes, the latch cannot reach the lock, and the clothes are not secured. Similarly, a mismatched substrate/effector pair such as a purine nucleotide with dATP would not be expected to allow the barrel to clamp and loop 2 to rearrange such that Arg298 can reach the substrate phosphate and thus form a ‘latched’ complex (Figure 10B). In the latter case, one would expect the mismatched substrate to be released from the enzyme, so that a complementary one can bind. Chemical logic tells us that allosteric regulation of specificity would require both low and high affinity substrate/effector-enzyme states; low affinity states to sample substrate/effector pairs, and high affinity states to capture the correct pair, recruit β_2_ (class I) or adenosylcobalamin (class II), and initiate catalysis (Figure 10). Although the molecular basis for the fivefold increase in binding affinity of β_2_ for α_2_ in *E. coli* class Ia RNR in the presence of bound substrate/effector pairs is not understood, the clamping of the barrel that we observe here may be part of the molecular explanation. The use of Arg298 as a molecular latch to secure the barrel in this closed state is beautiful in its simplicity. It is also consistent with both our structural and our biochemical data that show almost complete loss of activity in the Arg298Ala variant (Figure 9).

**Figure 11.**
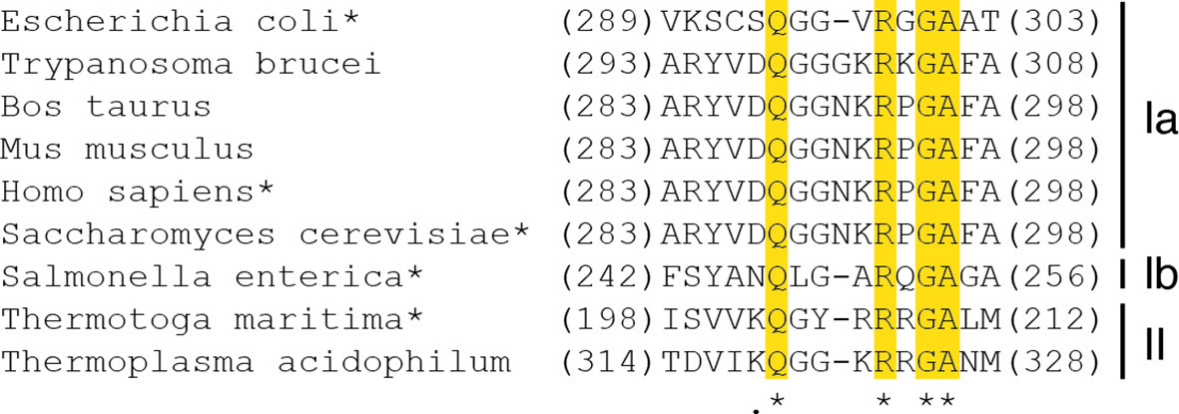
Structure-based sequence alignment of loop 2 residues of characterized class Ia, class Ib, and class II RNRs, with asterisk denoting RNRs for which structures are available. Absolutely conserved residues are starred and highlighted. Arg298 (*E. coli* numbering) stabilizes substrate binding and Gln294 stabilizes pyrimidine binding when dATP is bound to the specificity allosteric site. The eukaryotic RNRs have one additional residue inserted into the loop. Characterized monomeric class II RNRs are not included in this alignment. Beyond these characterized RNRs, sequence alignments are more challenging and conservation is less clear. Although Arg298 may be strictly conserved, Gln294 is unlikely to be. **DOI:** http://dx.doi.org/10.7554/eLife.07141.018

Though it is generally accepted that the rules of specificity regulation are conserved among class I, II, and III RNRs, this hypothesis is based on a relatively small number of characterized RNRs. Loop 2 is clearly an important player in specificity regulation, and yet it is not highly conserved, even among characterized RNRs (Figure 11). This being said, our structures show that backbone atoms of loop 2 make the vast majority of contacts to the substrate base and the effector base, limiting the number of residues that need to be strictly conserved to allow for the same molecular mechanism. Also, despite the fairly low sequence identities between the *E. coli* and the *T. maritima* α subunits (20.5%) and the *E. coli* and the *S. cerevisiae* α subunits (29.3%), a structural comparison shows that all three enzymes interact with the phosphate groups and ribose moieties of their substrates and effectors in similar ways (Table 4).

**Table 4.** Interactions that anchor the ribose and phosphate moieties of substrate and specificity effector molecules to RNRs (bound waters are not included) **DOI:** http://dx.doi.org/10.7554/eLife.07141.019

	*E. coli *(this work)	*S. cerevisiae *(PDB ID: 2CVX)	*T. maritima *(PDB ID: 1XJE)
**Substrates**			
NDP ribose 3'-OH H bonds to:	Asn437 Glu441	Asn426 Glu430	Asn320 Glu324
NDP ribose 2'-OH H bonds to:	Backbone carbonyl	Backbone carbonyl	Backbone carbonyl
NDP phosphates H bonds to:	Backbone amides Thr209 Ser625 Arg298	Backbone amides Ser202 Thr611 Arg293*	Backbone amides Ser91 -– Arg207**
**Effectors**			
dNTP ribose 3'-OH H bond to:	Asp232 His 275	Asp226 -–	Asp141 -–
dNTP phosphates H-bond to:	Backbone amides Arg262 Arg269	Backbone amides Arg256 Lys243 (from a different helix)	Backbone amides Arg171 Lys158 (from a different helix)

*Arg293 residue does not hydrogen bond to the substrate phosphate, but this observed lack of interaction may be due to crystal packing (see text).

**Arg207 hydrogen bonds to the substrate phosphate in the one structure (GDP/TTP) that has loop 2 fully modeled.

The only noteworthy difference is that Arg298, critical for reduction of all four NDPs in *E. coli*, directly contacts the β-phosphate of all four substrates. In contrast, none of the four *S. cerevisiae* RNR structures show a direct contact to the β-phosphate by the equivalent arginine, although one structure shows a through-water contact, and only one of the substrate/effector bound structures of *T. maritima* RNR (GDP/TTP) shows direct contact. This GDP/TTP-bound structure is the only *T. maritima* RNR structure in which loop 2 is fully ordered and it is remarkably similar to our *E. coli* structures (Figure 10H,I). All contacts made to GDP are the same: Arg side chain to β-phosphate, and loop 2 carbonyl to N1 and N2 of base (Figure 10H, 4C). The TTP contacts are not identical, but in both cases, the resulting loop 2 conformations allow the carbonyl of 299 (*E. coli* numbering) and 208 (*T. maritima* numbering) to ‘fall back’ to contact N1 and N2 of GDP.

Excitingly, the *T. maritima* class II RNR structures also reveal a movement of Gln294 in and out of the active site in response to effector binding. In particular, the GDP/TTP and CDP/dATP bound structures from *T. maritima* class II RNR, which have an ordered and semi-ordered loop 2, respectively, show that Gln203 (equivalent to Gln294 in *E. coli*) interacts directly with CDP when dATP is bound, and moves away from the active site when GDP/TTP are bound (Larsson et al., 2004). Unfortunately, many loop 2 residues are disordered and thus not modeled in the *T. maritima* structures with CDP/dATP, UDP/dATP, and ADP/dGTP, preventing further comparison to our *E. coli* structures.

In stark contrast to the behavior of Gln294 in *E. coli* and Gln203 in *T. maritima*, Gln288 in *S. cerevisiae* class Ia RNR is observed to point into the active site in all structures, regardless of which substrate/effector pairs are bound. Structural comparisons show that the protein does not pack as tightly around purine substrates when Gln points into the active site as it does when Gln is flipped away (Figure 10E,F compared with 10H,I). It is possible that this difference in Gln positioning in *S. cerevisiae*, and differences described above for Arg298 (*E. coli* numbering), indicate that the mechanism of allosteric regulation is not conserved among RNRs. However, it is also possible that these observed structural differences are a result of crystal packing variations or other deviations in how crystals were prepared. Additional structures of high-affinity RNR-substrate complexes will help to clarify the degree to which the roles of Gln294 and Arg298 are conserved across RNR species. Regardless of their exact roles in *S. cerevisiae* RNR, there are a number of studies that support the importance of these residues (Ahmad et al., 2012; Kumar et al., 2010; Kumar et al., 2011). In particular, when mutant RNR is the only RNR being expressed in *S. cerevisiae,* mutation of Arg293 (the Arg298 equivalent) to Ala is lethal, and mutation of Gln288 (the Gln294 equivalent) to Ala yields a severe S phase defect (Ahmad et al., 2012). Gln288Ala mutation in *S. cerevisiae* also leads to substantially elevated dGTP/dATP levels compared with dCTP/TTP levels when one compares wild-type *S. cerevisiae* expressing two wild-type RNRs with mutant *S. cerevisiae* expressing one wild-type RNR and one Gln288Ala mutant RNR (Kumar et al., 2010). Under the same experimental conditions, all four dNTPs are elevated by similar amounts (within 1–3%) for the Arg293Ala mutation in *S. cerevisiae* (Kumar et al., 2010). Although more studies are clearly needed to confirm or refute that the molecular basis of allosteric specificity regulation among RNRs is conserved, it is interesting to note that our in vitro data on *E. coli* (Figure 9) is consistent with the more severe phenotype in *S. cerevisiae* for Arg than Gln mutation and is consistent with the observation that mutation of Gln in *S. cerevisiae* elevates purines over pyrimidines whereas mutation of Arg does not show differential elevation.

In conclusion, the work presented here provides a unifying mechanism for substrate specificity regulation in the most studied RNR, the *E. coli* class Ia enzyme. Our structures show how each specificity effector is read out at a distal allosteric site and how that information is communicated to the active site where residues rearrange such that specific hydrogen bonds can be formed with the cognate substrate base. When an effector/substrate match is discovered, the barrel is clamped and latched in preparation for catalysis. Just as DNA replication and transcription take advantage of the unique hydrogen-bonding properties of each nucleotide base, enzymatic ribonucleotide reduction also employs these unique hydrogen-bonding properties for specificity regulation. Through an elegant set of protein rearrangements, *E. coli* RNR screens and selects its substrate from the four potential NDPs, ensuring appropriate pools of deoxynucleotides are available for DNA biosynthesis and repair.

## Materials and methods

### Effector and substrate preparation

For the enzyme assays, sodium salts of CDP, ADP, UDP, GDP, dATP, dGTP, and TTP were purchased from Sigma-Aldrich (St. Louis, MO) and dissolved into assay buffer (50 mM HEPES pH 7.6, 15 mM MgCl_2_, 1 mM ethylenediaminetetraacetic acid (EDTA)). The pH of each solution was slowly adjusted to 7–8 with NaOH, and the nucleotide concentrations were determined spectroscopically, using ε_271_ of 9.1 mM^-1^ cm^-1^ for CDP, ε_259_ of 15.4 mM^-1^ cm^-1^ for ADP, ε_262_ of 10.0 mM^-1^ cm^-1^ for UDP, ε_253_ of 13.7 mM^-1^ cm^-1^ for GDP, ε_259_ of 15.4 mM^-1^ cm^-1^ for ATP, ε_259_ of 15.2 mM^-1^ cm^-1^ for dATP, ε_253_ of 13.7 mM^-1^ cm^-1^ for dGTP, and ε_262_ of 9.6 mM^-1^ cm^-1^ for TTP. Preparation of nucleotides for crystallography was the same as described above except that 100 mM solutions of sodium salts of dATP, dGTP, and TTP were purchased from USB Corporation (Cleveland, OH) or Invitrogen (Carlsbad, CA).

### Protein preparation for crystallography

The α_2_ and β_2_ proteins were prepared as described (Salowe et al., 1987; Salowe and Stubbe, 1986). The concentrations of α_2_ and β_2_ were determined using ε_280_ of 189 and 131 mM^-1^cm^-1^, respectively; unless noted otherwise, all molar concentrations are dimer concentrations (i.e. α_2_ or β_2_). For all structures, hydroxyurea-inactivated β_2_ (met-β_2_) was used in place of active β_2_. Met-β_2_ was prepared from purified active β_2_ as described (Ando et al., 2011). α_2_ had a specific activity of 3800 nmol min^-1^ mg^-1^. Prior to hydroxyurea treatment, β_2_ had a specific activity of 7700 nmol min^-1^ mg^-1^ as determined by a coupled spectrophotometric assay (Ge et al., 2003).

### Crystallization

Crystals were grown using the hanging drop vapor diffusion technique by mixing 1 μL of protein (25 μM α_2_ and 50 μM met-β_2_ in 50 mM HEPES, 15 mM MgCl_2_, and 1 mM EDTA, pH 7.6, supplemented with 10 mM dATP and 5 mM DTT, 1% (w/v) isopropyl-β-thiogalactopyranoside) with 1 μL of precipitant solution and equilibrating over a reservoir of 500 μL of precipitant at room temperature (~25°C). For the CDP/dATP structure, the precipitant solution was 9.5% (w/v) PEG 3350, 100 mM MOPS pH 7.5, 250 mM Mg(CH_3_COO)_2,_ 25 mM MgCl_2_, and 5% (v/v) glycerol. For the UDP/dATP, ADP/dGTP, and GDP/TTP structures, the precipitant solution was 12% (w/v) PEG 3350, 100 mM MOPS pH 7.5, 300 mM Mg(CH_3_COO)_2_, 30 mM MgCl_2_, and 5% (v/v) glycerol. Freshly prepared drops were streak seeded with microcrystals grown under the same conditions with the exception of the MgCl_2_ concentration in the precipitant being 100 mM MgCl_2_. Streak seeding was used to obtain larger single crystals.

After 2 days of growth, crystals were transferred to a drop of soaking solution. For the CDP/dATP structure, dATP was co-crystallized, and CDP was soaked into the structure using a solution containing 10.5% (w/v) PEG 3350, 100 mM MOPS pH 7.5, 25 mM MgCl_2_, 250 mM Mg(CH_3_COO)_2_, 5% (v/v) glycerol, 5 mM DTT, and 10 mM CDP. For the GDP/TTP, UDP/dATP and ADP/dGTP structures, the soaking solutions were the same as for CDP except for having 13% (w/v) PEG 3350, and each nucleotide at 10 mM. Crystals were left in the soaking solution for 2 min and then cryoprotected. CDP-soaked crystals were looped through a solution of 12% (w/v) PEG 3350, 100 mM MOPS pH 7.5, 250 mM Mg(CH_3_COO)_2,_ 60 mM MgCl_2_ and 10%, 15%, and 20% (v/v) glycerol in succession and then plunged directly into liquid N_2_. GDP/TTP, UDP/dATP, and ADP/dGTP soaked crystals were looped through a solution of 14% (w/v) PEG 3350, 100 mM MOPS pH 7.5, 300 mM Mg(CH_3_COO)_2_, 100 mM MgCl_2_, and 10%, 15%, and 20% (v/v) glycerol in succession and then plunged directly into liquid N_2_.

### Structure solution and refinement

Diffraction data were collected at the Advanced Photon Source at Argonne National Laboratory. The CDP/dATP data set was collected on beamline 24ID-C at 100 K on an ADSC Q315 detector. The UDP/dATP, ADP/dGTP, and GDP/TTP data sets were collected on beamline 24ID-C at 100 K on a Pilatus 6M detector. All data were processed using HKL2000 (Otwinowski and Minor, 1997) (Table 2).

All four α_4_β_4_ complex structures soaked with substrates and effectors were solved to the full extent of the data resolution using the previously published 3.95-Å resolution structure (Zimanyi et al., 2012) with all nucleotides removed. *R*_free_ test sets were chosen to contain the same reflections across all data sets. For all structures, initial refinement was carried out in CNS 1.3 (Brünger et al., 1998) and later in Phenix (Afonine et al., 2012) with model building performed in COOT (Emsley et al., 2010). Refinement consisted of rigid body, simulated annealing, positional and individual B-factor refinement with no sigma cutoff. Loose non-crystallographic symmetry restraints were used throughout refinement. Simulated annealing composite omit maps generated in CNS 1.3 and Phenix were used to verify the models. All figures were made using the PyMOL Molecular Graphics System, version 1.5.0.4 (Schrödinger, LLC). Final refinement statistics are shown in Table 2. In addition to the substrate/specificity effector pairs bound in each structure, all structures have dATP or its hydrolysis product dADP in the allosteric activity site. In some cases, the high concentration of nucleotide used (10 mM) has resulted in more than one nucleotide molecule bound near this allosteric site.

### Calculation of difference distance matrix plots

Difference distance matrix plots were produced using the DDMP program from the Center for Structural Biology at Yale University (New Haven, CT). Residues 4 to 737 of chain A from each structure were used for the analysis.

### Mutagenesis and protein purification for activity assays

Gln294Ala-α_2_ and Arg298Ala-α_2_ were constructed from the previously published vector pET-nrdA (Minnihan et al., 2011) through QuikChange site-directed mutagenesis (Agilent, Santa Clara, CA) using the following primers from Integrated DNA Technologies (Coralville, IA): Gln294Ala forward primer (5'’-AAATCCTGCTCTGCGGGCGGTGTGC-3’), Gln294Ala reverse primer (5’-GCACACCGCCCGCAGAGCAGGATTT-3’), Arg298Ala forward primer (5’-CGTTGCCGCACCGCCAGCCACACCGC-3’), and Arg298Ala reverse primer (5’-GCGGTGTGGCTGGCGGTGCGGCAACG-3’). All mutations were confirmed by DNA sequencing performed by Genewiz (South Plainfield, NJ).

Expression and purification of N-terminal hexahistidine tagged *E. coli* wild-type α_2_ and N-terminal hexahistidine tagged mutant α_2_ variant proteins (Gln294Ala-α_2_ and Arg298Ala-α_2_) were carried out as previously described (Minnihan et al., 2011). Briefly, cells were resuspended in 40 mL of Buffer A (50 mM Tris pH 7.6, 300 mM NaCl, 1 mM TCEP), lysed by sonication, and clarified by centrifugation at 29,000 × g. Lysate was applied to a 5 mL HisTrap HP column (GE Healthcare Life Sciences, Pittsburg, PA), washed with Buffer A supplemented with 30 mM imidazole, and eluted with Buffer A supplemented with 300 mM imidazole. Protein was further purified on a Superdex 200 16/60 size exclusion column (GE Healthcare Life Sciences) and transferred to a final storage buffer of 20 mM HEPES 7.6, 100 mM NaCl, and 5% glycerol. A final yield of ~25–50 mg/L of culture for wild-type α_2_ is typical. The purification for the mutants was identical, with similar yields. All proteins were judged as purified to homogeneity by sodium dodecyl sulfate/polyacrylamide gel electrophoresis (SDS/PAGE), and their concentrations were determined using ε_280_ of 189 mM^-1^cm^-1^. Hexahistidine tags were not removed since previous studies showed that these tags on *E. coli* α_2_ do not significantly alter activity (Minnihan et al., 2011). Untagged *E. coli* β_2_ was purified as previously described (Salowe and Stubbe, 1986) and contained ~1.1 radicals per dimer as estimated by UV-visible spectroscopy of the Y_122_ radical (ε_411_ of 1760 mM^-1^ cm^-1^) after drop-line subtraction of the diferric cluster absorbance (Bollinger et al., 1995). Untagged *E. coli* β_2_ was exchanged into a storage buffer containing 50 mM HEPES 7.6 and 5% glycerol, and its concentration determined using ε_280_ of 131 mM^-1 ^cm^-1^.

For the coupled assay described below, *E. coli* thioredoxin reductase (TrxR) and *E. coli* thioredoxin (Trx) were prepared. The gene for TrxR was amplified from *E. coli* genomic DNA using primers with ends suitable for a second round of PCR to generate the plasmid borne gene in the pRham SUMO fusion vector (Lucigen, Middleton, WI). The primers, ordered from Integrated DNA Technologies, were: forward 5’-CGCGAACAGATTGGAGGTGGCACGACCAAACACAGTAAACTG-3’, and reverse 5’-GTGGCGGCCGCTCTATTATTTTGCGTCAGCTAAACCATCGAG-3’. DNA sequencing performed by Genewiz was used to confirm the sequence of the resulting construct. This construct contains a hexahistidine tag and SUMO protein fusion at the N-terminus under a rhamnose promoter. The resulting protein was expressed according to the plasmid manufacturer protocol (Lucigen) and purified as described above for hexahistidine-tagged α_2_, using the same buffers. The amount of flavin cofactor was quantified by absorption at 440 nm (Thelander, 1967; Gleason et al., 1990), and a specific activity of 38,200 nmol min^-1^ mg^-1^ was determined by a coupled assay with 30 µM TR and 150 µM Ellman’s reagent (5,5'-dithiobis[2-nitrobenzoic acid]) (Pierce, Rockford, IL) (Luthman and Holmgren, 1982). The final concentration for assays (0.5 µM) was determined by flavin concentration as the as-expressed protein was only 60% loaded with flavin as determined by the A280/A440 ratio. The resulting protein behaved identically in assays as untagged TrxR purified from an overexpressing *E. coli* strain (Russel and Model, 1985). *E. coli* Trx was purified as previously described (Chivers et al., 1997).

### Activity assays

Activity assays for wild-type α_2_ and the Gln294Ala and Arg298Ala mutants were performed using a continuous, coupled, spectrophotometric assay monitoring the consumption of NADPH by the Trx/TrxR system (Ge et al., 2003). All experiments were performed on a Cary Bio300 spectrometer (Agilent) with data analysis performed using the Cary WinUV Kinetics program (Agilent) and Microsoft Excel. The assay buffer consisted of 50 mM HEPES pH 7.6, 15 mM MgCl_2_, 1 mM EDTA, and the following substrate and effector concentrations were used: 1 mM CDP and 3 mM ATP (control for presence of activity), 1 mM CDP and 175 μM dATP (control for inactivation by dATP), 1 mM ADP and 120 μM dGTP, 1 mM GDP and 250 μM TTP, and 1 mM CDP/UDP and 1 μM dATP. Substrate, effector, *E. coli* Trx (30 μM), *E. coli* TrxR (0.5 μM), and NADPH from Sigma-Aldrich (200 μM) were mixed in assay buffer, and the reaction was initiated by the addition of α_2_ (0.1 μM) and wild-type β_2_ (1 μM) to a final volume of 120 μL. The decrease in NADPH absorbance at 340 nm was monitored over 1 min. The basal level of NADPH oxidation was monitored over 30 s prior to the addition of enzyme.

## Additional files

### Major datasets

The following datasets were generated:

Chen P.Y.-T, Zimanyi C.M., Drennan C.L.,2015,Crystal structure of the dATP inhibited E. coli class Ia ribonucleotide reductase complex bound to CDP and dATP at 2.97 Angstroms resolution,http://www.rcsb.org/pdb/explore.do?structureId=5CNS,5CNS

Chen P.Y.-T, Zimanyi C.M., Drennan C.L.,2015,Crystal structure of the dATP inhibited E. coli class Ia ribonucleotide reductase complex bound to UDP and dATP at 3.25 Angstroms resolution,http://www.rcsb.org/pdb/explore/explore.do?structureId=5CNT,5CNT

Chen P.Y.-T, Zimanyi C.M., Drennan C.L.,2015,Crystal structure of the dATP inhibited E. coli class Ia ribonucleotide reductase complex bound to ADP and dGTP at 3.40 Angstroms resolution,http://www.rcsb.org/pdb/explore/explore.do?structureId=5CNU,5CNU

Chen P.Y.-T, Zimanyi C.M., Drennan C.L.,2015,Crystal structure of the dATP inhibited E. coli class Ia ribonucleotide reductase complex bound to GDP and TTP at 3.20 Angstroms resolution,http://www.rcsb.org/pdb/explore/explore.do?structureId=5CNV,5CNV

The following previously published datasets were used:

Eriksson M, Uhlin U, Ramaswamy S, Ekberg M, Regnstrom K, Sjoberg BM, Eklund H,1997,Ribonucleotide reductase R1 protein with substrate, GDP and effector dTTP fro Escherichia coli,http://www.rcsb.org/pdb/explore.do?structureId=4r1r,4R1R

Eriksson M, Uhlin U, Ramaswamy S, Ekberg M, Regnstrom K, Sjoberg BM, Eklund H,1997,Ribonucleotide reductase R1 protein with AMPPNP occupying the activity site from Echerichia coli,http://www.rcsb.org/pdb/explore/explore.do?structureId=3R1R,3R1R

Ando N, Brignole EJ, Christina M Zimanyi, Michael A Funk, Yokoyama K, Asturias FJ, Stubbe J, Catherine L Drennan,2011,Crystal structure of the dATP inhibited E. coli class Ia ribonucleotide reductase complex,http://www.rcsb.org/pdb/explore/explore.do?structureId=3UUS,3UUS

Nordlund P, Eklund H,1993,Structure and function of the Escherichia coli ribonucleotide reductase protein R2,http://www.rcsb.org/pdb/explore/explore.do?structureId=1RIB,1RIB

Xu H, Faber C, Uchiki T, Fairman JW, Racca J, Dealwis C,2006,Structures of Yeast Ribonucleotide Reductase I,http://www.rcsb.org/pdb/explore/explore.do?structureId=2CVX,2CVX

Larsson K-M, Jordan A, Eliasson R, Reichard P, Logan DT, Nordlund P,2004,Structural mechanism of allosteric substrate specificity in a ribonucleotide reductase: dTTP-GDP complex,http://www.rcsb.org/pdb/explore/explore.do?structureId=1XJE,1XJE
